# Identification of a two-SNP *PLA2R1* Haplotype and *HLA-DRB1* Alleles as Primary Risk Associations in Idiopathic Membranous Nephropathy

**DOI:** 10.1038/s41598-018-33612-7

**Published:** 2018-10-22

**Authors:** Khun Zaw Latt, Kenjiro Honda, Myo Thiri, Yuki Hitomi, Yosuke Omae, Hiromi Sawai, Yosuke Kawai, Shunsuke Teraguchi, Kazuko Ueno, Masao Nagasaki, Akihiko Mabuchi, Hajime Kaga, Atsushi Komatsuda, Katsushi Tokunaga, Eisei Noiri

**Affiliations:** 1 Department of Human Genetics, Graduate School of Medicine, The University of Tokyo, Tokyo, Japan; 2 Department of Nephrology and Endocrinology, The University of Tokyo Hospital, Tokyo, Japan; 3 Department of Hemodialysis and Apheresis, The University of Tokyo Hospital, Tokyo, Japan; 4 Department of Hematology, Nephrology, and Rheumatology, Graduate School of Medicine, Akita University, Akita, Japan; 5 Department of Integrative Genomics, Tohoku Medical Megabank Organization, Tohoku University, Sendai, Japan

## Abstract

The associations of single nucleotide polymorphisms (SNPs) in *PLA2R1* and *HLA-DQA1*, as well as HLA-DRB1*15:01-DQB1*06:02 haplotype with idiopathic membranous nephropathy (IMN) is well known. However, the primary associations of these loci still need to be determined. We used Japanese-specific SNP genotyping array and imputation using 2,048 sequenced Japanese samples to fine-map *PLA2R1* region in 98 patients and 413 controls. The most significant SNPs were replicated in a separate sample set of 130 patients and 288 controls. A two-SNP haplotype of intronic and missense SNPs showed the strongest association. The intronic SNP is strongly associated with *PLA2R1* expression in the Genotype-Tissue Expression (GTEx) database, and the missense SNP is predicted to alter peptide binding with HLA-DRB1*15:01 by the Immune Epitope Database (IEDB). In *HLA* region, we performed relative predispositional effect (RPE) tests and identified additional risk alleles in both *HLA-DRB1* and *HLA-DQB1*. We collapsed the risk alleles in each of *HLA-DRB1* and *HLA-DQB1* into single risk alleles. Reciprocal conditioning of these collapsed risk alleles showed more residual significance for *HLA-DRB1* collapsed risk than *HLA-DQB1* collapsed risk. These results indicate that changes in the expression levels of structurally different PLA2R protein confer risk for IMN in the presence of risk *HLA-DRB1* alleles.

## Introduction

Idiopathic membranous nephropathy (IMN) or primary membranous nephropathy is an autoimmune kidney disease and one of the most common causes of primary nephrotic syndrome in adults. IMN is an antibody-mediated autoimmune disease specific to kidneys, a primary glomerular disease distinct from secondary membranous nephropathy, which manifests as an associated complication of other systemic diseases (eg. SLE, Lung Cancer & Hepatitis B). The major auto-antigen in IMN has been identified as the M-type phospholipase A_2_ receptor (PLA_2_R), and anti-PLA2R antibodies were found in the sera of 75% of IMN patients^1,2^.

The first genome-wide association study (GWAS) of IMN was done in the European population, and it identified associations with single nucleotide polymorphisms (SNPs) in *PLA2R1* on chromosome 2 and the *HLA-DQA1* region on chromosome 6^3^. Top SNPs in *PLA2R1* (rs4664308) and *HLA-DQA1* (rs2187668) have been widely replicated in different populations^4–6^. In the Japanese population, a previous candidate-gene study reported rs3749119 in the 5′ untranslated region (5′ UTR) as the strongest association in *PLA2R1*, and association of a haplotype of *HLA*-DRB1*15:01 and HLA-DQB1*06:02 in the *HLA* region^7^.

However, the previous Japanese study only genotyped 15 SNPs in *PLA2R1* and may not have comprehensively covered the whole gene region. And despite all these reported SNP associations in *PLA2R1*, the primary SNP associations as well as the underlying mechanism of the risk has not been discovered.

In the *HLA* region, one European study reported strong association with the haplotype of the HLA-DRB1*03:01, HLA-DQA1*05:01 and HLA-DQB1*02:01 alleles which individually showed similar levels of association and are in strong linkage disequilibrium (LD)^8^. In Japanese, HLA-DRB1*15:01 and HLA-DQB1*06:02 alleles are also in very strong LD and showed similar associations. The previous Japanese study also reported the association of the HLA-DRB1*15:01-DQB1*06:02 haplotype and could not distinguish the primary risk allele^7^.

In this study, to determine the primary risk associations in these loci and to potentially elucidate the disease-causing mechanisms, we examined the IMN associations in *PLA2R1* and *HLA* regions comprehensively by a using genome-wide SNP array, robust statistical methods and functional annotations. Our results indicate that a two-SNP *PLA2R1* haplotype, which causes changes in both structure and expression levels of PLA2R protein, and *HLA-DRB1* alleles are the primary causal associations for IMN.

## Results

### Fine-mapping *PLA2R1* for primary association

#### Population-specific SNP array and imputation

We genotyped 98 IMN cases and 413 healthy controls using Affymetrix Japonica genotyping array, and found that the top associated SNPs in *PLA2R1* were similar to the previous Japanese study and the European GWAS. The most significant association was rs16844715, closely followed by rs4665147, which is in complete LD (r^2^ = 1) with the European GWAS top SNP, rs4664308, and then by rs17830904, which is also in complete LD with rs35771982, which is the second top associated SNP in the previous Japanese study (Table 1). We performed imputation of the ungenotyped SNPs using 2,048 sequenced Japanese samples from the Tohoku Medical Megabank Organization (ToMMo) as the reference panel but the top association signals were essentially the same and we did not detect any other signal beyond the LD coverage of the top genotyped SNPs (Supplementary Table S1).

**Table 1:** Association results of SNPs in *PLA2R1* with P < 10^−5^ in the initial genome-wide genotyping results. (98 IMN vs. 413 controls).

SNP	Alleles	MAF	OR (95% CI)	1/OR	P	LD (r^2^)
	(Minor/Major)	(Case/Control)				
rs16844715	T/C	0.33/0.54	0.41 (0.29–0.57)	2.44	5.53 × 10^−8^	
rs4665147	G/A	0.24/0.43	0.41 (0.29–0.59)	2.42	8.83 × 10^−7^	1 with rs4664308
rs4664308	G/A	0.24/0.43	0.42 (0.3–0.60)	2.38	1.19 × 10^−6^	(EUR GWAS top hit)
rs17831161	G/A	0.24/0.42	0.42 (0.3–0.61)	2.36	1.63 × 10^−6^	
rs17830904	G/A	0.26/0.44	0.45 (0.32–0.64)	2.24	4.60 × 10^−6^	1 with rs3749117 & rs35771982
rs6759924	A/G	0.19/0.36	0.41 (0.28–0.61)	2.43	5.10 × 10^−6^	0.64 with rs4664308
rs55977890	T/C	0.31/0.49	0.47 (0.34–0.65)	2.14	5.23 × 10^−6^	
rs10929956	C/T	0.27/0.44	0.46 (0.33–0.65)	2.18	7.68 × 10^−6^	
rs35771982	C/G	0.26/0.44	0.45 (0.32–0.65)	2.21	9.57 × 10^−6^	
rs7601374	C/T	0.30/0.48	0.48 (0.34–0.67)	2.1	9.99 × 10^−6^	

rs4664308 is the top SNP and rs3749117 is the second top SNP in European GWAS.

We chose the top four SNPs from our initial association results: rs16844715, rs4664308, and rs35771982 as the strongest association signals, and rs3749119, which is the strongest association in the previous Japanese study for comparison, and replicated these four SNPs in an independent sample set of 130 IMN cases and 288 healthy controls. In the combined analysis of all samples, all 4 SNPs showed similar degrees of association, and rs4664308 became the strongest signal (OR = 0.39, P = 8.07 × 10^−14^), followed by rs3749119, rs35771982, and rs16844715 (Table 2).

**Table 2:** The association results of top *PLA2R1* SNPs in the combined dataset (222 IMN vs. 701 controls).

SNP	Alleles	MAF	OR (95% CI)	1/OR	P-value	Annotation	LD
	(Minor/Major)	(case/control)					
rs4664308	G/A	0.22/0.42	0.39 (0.31–0.51)	2.54	8.07 × 10^−14^	Intron 1	r^2^ = 0.94 in Japanese
rs3749119	T/C	0.22/0.41	0.4 (0.31–0.52)	2.49	3.56 × 10^−13^	5′ UTR	
rs35771982	C/G	0.23/0.42	0.41 (0.32–0.53)	2.41	8.85 × 10^−13^	Missense	
rs16844715	T/C	0.33/0.54	0.43 (0.34–0.54)	2.34	6.34 × 10^−14^	Intron 1	

rs4664308 is the strongest association followed by rs3749119 which is essentially the same signal as the top SNP because of the very high LD. This makes rs35771982 the next strongest association signal.

**Figure 1:**
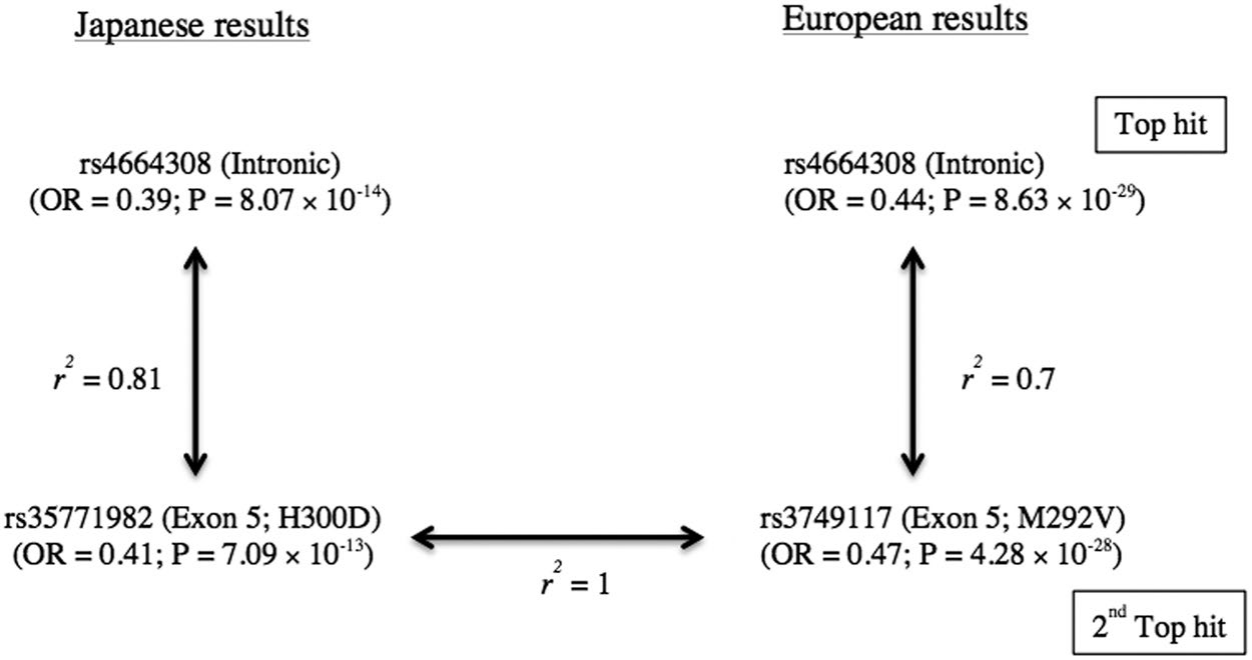
Comparison between current Japanese results and European GWAS results. The LD values between the intronic and missense *PLA2R1* SNPs are from case-control combined data of European GWAS and the current study. The r^2^ value between rs35771982 and rs3749117 is 1 for Asian and 0.98 for European (HaploReg database v4.1).

The SNP with the second strongest association, rs3749119, is located in the 5′ UTR and is in very strong LD (r^2^ = 0.94) with the top SNP in our combined dataset, making it essentially the same signal as the top SNP. The next SNP, rs35771982, is located in exon 5 and has an LD value (r^2^) with the top SNP of 0.81 (Fig. 1).

#### Comparison of top associated SNPs between populations

We recognized, here, a similar pattern between the Japanese and European results. In the European GWAS, the top SNP (rs4664308) and the second top SNP (rs3749117) from exon 5 showed a similar degree of association in both effect size and significance, although the LD between them is rather moderate (r^2^ = 0.7)^3^. In Asian, both rs35771982 and rs3749117 are located in exon 5 of *PLA2R1* gene and are in complete LD (r^2^ = 1) (HaploReg v4.1. 1000 genome), and rs35771982 also showed a similar level of association with rs4664308 in this study (Fig. 1).

#### Haplotype relationship between the top two SNPs

**Figure 2:**
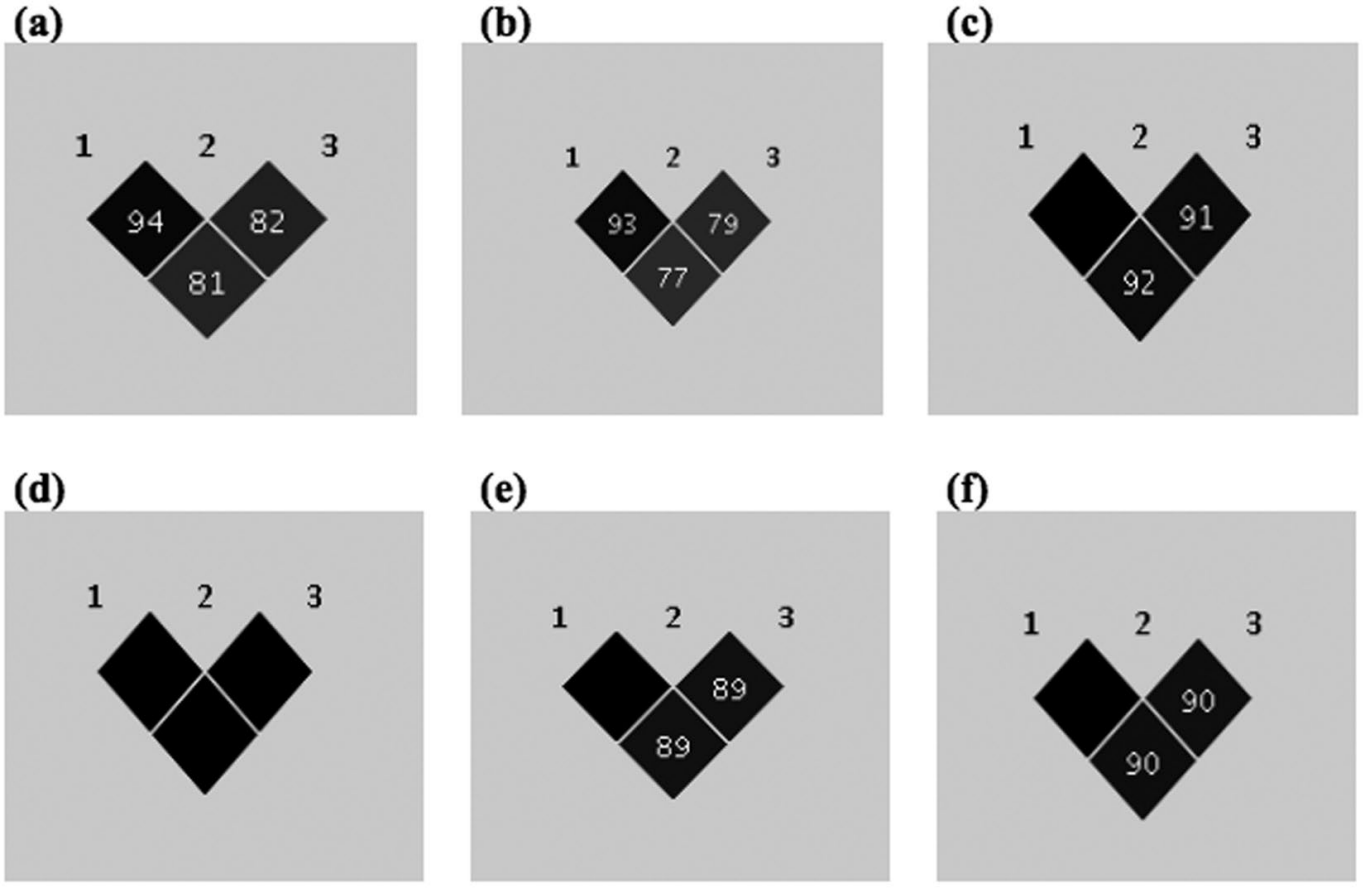
Dissecting LD among top *PLA2R1* SNPs. 1 = intronic (rs4664308), 2 = 5′UTR SNP (rs3749119), 3 = exon 5 missense SNP (rs35771982). LD (r^2^) values in (**a**) all case-control combined data (**b**) all control samples (**c**) all IMN case samples (**d**) 50 IMN samples from the University of Tokyo Hospital (**e**) 42 IMN samples from Akita University and (**f**) 130 IMN samples from the BioBank Japan.

We further investigated the relationship of these 2 SNPs in detail. The r^2^ value between these two SNPs is 0.81 in our case-control combined dataset. Upon conditioning rs4664308, rs35771982 showed no residual significance and *vice versa* (Supplementary Table S2), meaning that there is no significant independent effect between these 2 SNPs. We then further dissected the LD structure of these 2 SNPs in patients and controls separately, and found that r^2^ is only 0.77 in controls and 0.92 in patients. We also checked the r^2^ values in each IMN dataset separately and the values were found to be consistently high in all IMN datasets (1 for samples from the University of Tokyo Hospital, 0.9 for samples from BioBank Japan and 0.89 for samples from Akita University) (Fig. 2).

Because of this higher LD between the two SNPs only in patients, we performed haplotype tests. The haplotype of the risk allele G of rs35771982 and the risk allele A of rs4664308 showed stronger association than the individual SNPs (OR = 2.68; P = 3.76 × 10^−15^) (Table 3). It is also stronger than the SNP associations in the recessive model (Supplementary Table S3) and also in interaction with HLA-DRB1*15:01 positivity status (Supplementary Table S4).

**Table 3:** Two SNP haplotype analysis of intronic (rs4664308) and missense (rs35771982) (upper table) and three SNP haplotype analysis including the 5′ UTR SNP (rs3749119) (lower table). (222 IMN vs. 701 controls).

Risk Haplotype	Frequency (case/control)	OR (95% CI)	P
**rs35771982-rs4664308 (2 SNPs)**			
GA	0.765/0.551	2.68 (2.1–3.42)	3.76 × 10^−15^
**rs35771982-rs4664308-rs3749119 (3 SNPs)**			
GAC	0.767/0.559	2.7 (2.11–3.44)	1.15 × 10^−14^

Two-SNP and three-SNP haplotypes are not different because 5′UTR SNP is the almost the same signal as the intronic SNP.

This stronger association of the haplotype than individual SNPs suggests two possibilities. The first is that there could still be a hidden causal variant with stronger effect size, and this haplotype is just tagging that variant better than the individual SNPs. However, reportedly in the European population, all 30 exons of *PLA2R1* were sequenced in IMN patients and no stronger association was found^9^. Moreover, the results of imputation using 2,048 sequenced Japanese samples did not find any stronger variant.

This led us to the second possibility, that the component SNPs of the haplotype themselves could be responsible for disease risk. The haplotype has two SNPs, which could correspond to two different components – one intronic, which could possibly regulate gene expression levels, and the other one missense, causing a change in protein structure. To test this hypothesis, we tried to get more information about the functions of these SNPs.

#### Functional annotations and predictions

To evaluate the functional significance of the SNPs in the haplotype, we checked the expression quantitative trait loci (eQTL) information for the intronic and 5′ UTR SNPs in the GTEx database^10^ and found that both are strongly associated with *PLA2R1* expression in multiple tissues (Table 4 & Supplementary Fig. S1). The intronic SNP was found to be in high LD (r^2^ ≥ 0.95) with the strongest eQTL SNPs with positive effect sizes in six tissue/cell types (subcutaneous adipose tissue, transformed fibroblasts, esophageal mucosa, sigmoid colon, skeletal muscle and spleen), and the 5′UTR SNP itself is the strongest eQTL SNP in three other tissues (tibial nerve, esophageal muscularis and atrial appendage of heart) (Supplementary Table S5). These two SNPs were also found to be significant eQTL SNPs for *PLA2R1* expression in microdissected glomerular tissue in NephQTL database (β = −0.15, P = 0.044 for rs4664308 & β = **−**0.18, P = 0.025 for rs3749119)^11^.

**Table 4:** Expression quantitative trait loci (eQTL) information for rs4664308 (intronic) and rs3749119 (5′ UTR) SNPs from Genotype-Tissue expression (GTEx) database.

Tissue	rs4664308 (Intronic)		rs3749119 (5′ UTR)	
	Effect Size (beta)	P-Value	Effect Size (beta)	P-Value
Adipose - Subcutaneous	0.27	3.8 × 10^−11^	0.28	2.7 × 10^−10^
Heart – Atrial Appendage	0.21	3 × 10^−6^	0.29	2 × 10^−8^
Cells-Transformed fibroblasts	0.26	1.7 × 10^−25^	0.28	3 × 10^−23^
Colon - Sigmoid	0.41	4.7 × 10^−10^	0.39	8.1 × 10^−9^
Esophagus - Mucosa	0.39	9.8 × 10^−26^	0.40	2.4 × 10^−22^
Esophagus - Muscularis	0.24	3.5 × 10^−10^	0.28	1.9 × 10^−11^
Muscle - Skeletal	0.29	2.4 × 10^−17^	0.29	1.5 × 10^−15^
Nerve - Tibial	0.20	6.8 × 10^−15^	0.22	3.7 × 10^−16^
Adrenal	0.36	5.2 × 10^−7^	0.42	3.3 × 10^−9^
Thyroid	0.20	2 × 10^−7^	0.22	4.3 × 10^−8^

Both are strongly associated with *PLA2R1* expression levels in multiple tissues.

For the coding SNP, we checked how the missense SNPs from both Japanese and European studies could potentially change the function of the protein using PolyPhen2 software^12^. Both SNPs are predicted to be benign by PolyPhen2, suggesting that they would not interfere with PLA2R function. Because the haplotype also showed strong interaction with HLA-DRB1*15:01, we tried to predict the effect of these missense SNPs on binding with the protein encoded by HLA-DRB1*15:01 using the IEDB database. The major alleles (G for rs35771982 and T for rs3749117) are risk alleles for both SNPs and encode histidine and methionine, which are risk amino acids, and the minor alleles (C for both) cause missense changes to non-risk amino acids, aspartate and valine, respectively.

The IEDB database predicts the binding affinity for overlapping peptides of PLA2R protein with protein encoded by HLA-DRB1*15:01 (Supplementary Table S6). The lower the percentile rank, the stronger the binding and *vice versa*. Peptides from risk and non-risk PLA2R proteins show generally similar percentile ranks until they include amino acid positions 292 to 300, which corresponds to rs3749117 and rs35771982, in which the risk peptides show obviously lower percentile rankings than their non-risk counterparts, suggesting that the risk peptides including these positions bind with stronger affinity to HLA-DRB1*15:01 protein.

### Primary associations in the *HLA* region

#### *HLA* SNP associations in Japanese IMN

From the initial dataset with genome-wide genotype data, the strongest SNP association in the *HLA* region was rs9268978 (OR = 3.52; P = 4.67 × 10^−10^) and after imputation the strongest SNP was rs9271147 (OR = 3.71; P = 6.63 × 10^−11^) (Supplementary Table S7 and S8). The top *HLA* SNP from the European GWAS, rs2187668, which is also a well-known tag SNP for HLA-DRB1*03:01, was clearly not significant (OR = 1.17; P = 0.61) (Supplementary Table S9). However, assuming that the classical *HLA* alleles are biologically more relevant to IMN than SNPs in the HLA region, we focused on classical *HLA* alleles to find the primary risk associations.

#### Relative predispositional effect (RPE) tests of *HLA-DRB1* and *HLA-DQB1* alleles

The previous Japanese study reported the haplotype of HLA-DRB1*15:01 and HLA-DQB1*06:02 to be strongly associated with IMN^7^. When a disease is associated with more than one allele of a gene, the strong association of one allele can cause misleading deviations in the frequencies of other alleles that are also associated, but to a weaker degree^13^. To look for additional risk alleles in both genes, we performed RPE tests that remove the strongest associated allele from each gene and repeat association in the remaining alleles (see Methods). RPE tests sequentially detected DRB1*14:54 and DRB1*11:01 alleles in *HLA-DRB1* gene, and DQB1*05:02 and DQB1*03:01 in *HLA-DQB1* gene (Table 5).

**Table 5:** RPE test results for *HLA-DRB1* and *HLA-DQB1* alleles. Each allele in each gene is the result of association after removing all the significant alleles listed above. (222 IMN vs. 701 Controls).

*HLA-DRB1* Alleles	IMN (2n = 428)		Control (2n = 1226)		OR	P	RPE		
	No.	%	No.	%	(95% CI)		OR (95% CI)	P	Pc
DRB1*15:01	91	21.26%	100	8.16%	3.04 (2.24–4.15)	2.80 × 10^−13^	—	—	—
DRB1*14:54	22	5.14%	38	3.10%	1.69 (0.99–2.9)	0.0519	2.5 (1.43–4.37)	0.00094	0.0188
DRB1*11:01	20	4.67%	31	2.53%	1.89 (1.07–3.35)	0.027	2.61 (1.39–4.91)	0.002	0.04
DRB1*14:05	20	4.67%	25	2.04%	2.35 (1.29–4.28)	0.0039	2.49 (1.18–5.22)	0.013	ns
***HLA-DQB1*** **Alleles**	**IMN (2n** = **427)**		**Control (2n = 1226)**		**OR**	**P**	**RPE**		
	**No**.	**%**	**No**	**%**	**(95% CI)**		**OR (95% CI)**	**P**	**Pc**
DQB1*06:02	84	19.67%	96	7.83%	2.88(2.1–3.96)	1.33 × 10^–11^	—	—	—
DQB1*05:02	18	4.22%	22	1.79%	2.41 (1.28–4.54)	0.0051	3.33 (1.69–6.57)	0.00024	0.00288
DQB1*03:01	66	15.46%	141	11.50%	1.41 (1.03–1.93)	0.0334	1.71 (1.19–2.46)	0.0032	0.0352
DQB1*05:03	22	5.15%	48	3.92%	1.33 (0.79–2.24)	0.27	1.83 (0.93–3.61)	0.078	ns

Pc = P-value adjusted for the number of *HLA* alleles with frequency in control group more than 0.5%.

ns = non-significant.

#### Collapsing risk alleles in each gene to differentiate the primary associations

Although DRB1*15:01 and DQB1*06:02 are in very high LD in Japanese, we assumed that the LD between the new alleles of these two genes might not necessarily be high. Therefore, considering that all alleles in each of *HLA-DRB1* or *HLA-DQB1* confer risk, we collapsed the alleles in each gene and projected them into single risk scores of *HLA-DRB1* and *HLA-DQB1* collapsed risk. In case-control association, the *DRB1* collapsed risk was stronger than the *DQB1* collapsed risk, and the difference was more prominent in the dominant model, which is the strongest of all models (Supplementary Table S10).

To distinguish the primary association between these two genes, we then performed reciprocal conditional tests on each of these collapsed risks. Upon conditioning the *DRB1* collapsed risk, the *DQB1* collapsed risk showed residual significance at P-value 0.005, and conditioning the *DQB1* collapsed risk gives the *DRB1* collapsed risk a significance of P-value 0.0001. Since their association is strongest in the dominant model, we did conditional tests in the dominant model and the difference in residual significance became more obvious. The residual P-value for the *DRB1* collapsed risk became 1.49 × 10^−5^ while that for the *DQB1* collapsed risk became only 0.012 (Table 6), suggesting that *HLA-DRB1* alleles are more likely to be the primary associations. We also evaluated the interaction between new *DRB1* alleles and the *PLA2R1* haplotype and found that both DRB1*14:54 and DRB1*11:01 showed interaction effects with the haplotype, albeit not as strongly as with DRB1*15:01 (Supplementary Table S11).

**Table 6:** Reciprocal conditional tests on *HLA-DRB1* and *HLA-DQB1* collapsed risk alleles in additive and dominant models. (222 IMN vs. 701 Controls).

Allele	A1	TEST	Samples	OR	P
**Additive Model**					
**Conditioning on DRB1_collapsed_risk**					
DQB1_collapsed_risk	P	Additive model	826	1.63	0.0051
**Conditioning on DQB1_collapsed_risk**					
DRB1_collapsed_risk	P	Additive model	826	2.08	0.00013
**Dominant Model**					
**Conditioning on DRB1_collapsed_risk**					
DQB1_collapsed_risk	P	Dominant model	826	1.72	0.012
**Conditioning on DQB1_collapsed_risk**					
DRB1_collapsed_risk	P	Dominant model	826	2.53	1.49 × 10^−5^

## Discussion

Despite the strong association of *PLA2R1* and *HLA* loci with IMN in many populations, the primary causal associations in both loci are still unclear because of the strong LD among variants and alleles in each locus. To address the need for understanding the primary causal associations in both loci, we performed comprehensive fine-mapping of both regions using a population-specific SNP array, a large reference panel for imputation and robust statistical methods to pinpoint the most likely and strongest primary associations.

The strong association of the two-SNP haplotype in *PLA2R1*, and the fact that there is no independent effect between the two SNPs, indicate the importance of the dual risk of both structural and expression-level changes in the PLA2R protein in this disease. Because of the strong LD (r^2^ ≥ 0.95) with the strongest eQTL SNPs with positive effects in multiple tissues (Supplementary Table S5), the intronic SNP (rs4664308) is considered to represent the strongest regulatory effect of the causal regulatory SNP, which could be one of the SNPs in high LD with the intronic SNP (Supplementary Table S12). The risk allele (major allele) of the intronic SNP corresponds to constitutively lower gene expression levels (Supplementary Fig. S4). It is possible that the expression levels may be changed (increased in this case) by such conditions as inflammation or exposure to foreign antigens and pollutants, and such increase in the level of structurally altered protein may provoke immunity in the presence of risk *HLA* alleles.

The missense SNPs in exon 5, rs3749117 and rs35771982, are the only two missense SNPs causing amino acid changes among all of the SNPs in high LD (r^2^ > 0.9) (Supplementary Table S13). They are both located in C-type lectin like domain 1 (CTLD1). These two SNPs are in complete LD (r^2^ = 1) in both Japanese and European populations (HaploReg v4.1. 1000 genome), making it difficult to statistically distinguish which one is causal or if both are important for disease risk. In 2015, two independent groups reported the immunodominant epitope PLA2R protein for the binding of autoantibodies. Anti-PLA2R antibodies from patients’ sera specifically recognized the complex consisting of the cysteine-rich (CysR), fibronectin-like type II (FnII) and C-type lectin-like domain 1 (CTLD 1) domains in PLA2R^14,15^.

These SNPs were predicted to cause no serious effects on protein function by PolyPhen2, but to bind with stronger affinity to DRB1*15:01. T-cell epitope prediction by a recent study using the SYFPEITHI database also found that a peptide in the CTLD 1 domain containing both missense amino acid positions is a possible epitope presented by DRB1*15:01, although they did not find the amino acid substitution at M292V to have influence on presentation modeling^16^. However, these prediction methods for MHC-peptide binding were reported to be inaccurate, and these prediction results should be confirmed by HLA-peptide binding assays.

Further functional studies should be aimed at evaluating the role of regulatory and missense SNPs in disease pathogenesis. There is also a recent report of the relationship between long-term exposure to air pollution and membranous nephropathy in China^17^. It is possible that exposure to pollutants and foreign antigens increases the intrarenal expression of structurally altered PLA2R protein that can be more easily presented to CD4^+^ T cells by specific risk *HLA* allele products or that can mimic the conformational structure of a foreign antigen which is the target of antibodies^14,18^.

For the *HLA* region, the European GWAS SNP, rs2187668 in *HLA-DQA1* gene, was found to completely lack significance. This is possible because this SNP is a well-known tag SNP for the HLA-DRB1*03:01 allele in northern European populations^19^, and the haplotype B*08:01-DRB1*03:01*DQA1*05:01-DQB1*02:01 is highly conserved and associated with several autoimmune diseases^8,20,21^. This particular allele and haplotype is absent in the Japanese population, and this explains the lack of significant association with rs2187668 in this study.

The previous Japanese study reported only HLA-DRB1*15:01 and HLA-DQB1*06:02 as risk alleles and haplotype in IMN. The haplotype DRB1*15:01-DQB1*06:02 is a common haplotype in the Japanese population and is also a risk for autoimmune diseases, including multiple sclerosis, systemic lupus erythematosus, narcolepsy and idiopathic pulmonary fibrosis^22–30^. The LD between these two alleles is very high and it is difficult to confidently decide which one is the primary association from statistical methods alone, and these studies only reported the haplotype association.

In this study, using RPE tests, we were able to find additional risk alleles in both *HLA-DRB1* and *HLA-DQB1* genes that could normally be masked by the strong associations of HLA-DRB1*15:01 and HLA-DQB1*06:02. Collapsing these alleles and reciprocal conditioning suggest that *HLA-DRB1* alleles are more likely to be primary. This is also consistent with a recent Chinese report in which HLA-DRB1*15:01 is the strongest *HLA* allele in Chinese IMN patients^16^.

The combined effect size of *PLA2R1* haplotype recessive state and HLA-DRB1*15:01 positivity status is relatively strong (OR = 16.13) (Supplementary Table S4) compared to the effect sizes of risk alleles in other complex diseases. This strong effect size may have some potential applications in genetic screening or prediction of disease risk in the general populations. It may also be useful in the diagnosis of *PLA2R1*-mediated IMN, together with renal biopsy and anti-PLA2R antibodies, and in the precision management of the disease if there is any specific disease course or treatment response associated with this disease subgroup.

Like most of the GWAS and genetic SNP association studies, the findings in this study are only limited to disease risk loci and the causal variants. For application of this genotype information to clinical management, it is necessary to explore further correlations with disease phenotypes, such as severity at onset, anti-PLA2R titers, response to immunosuppressive therapy and long-term renal prognosis. A recent study has demonstrated that epitope spreading of anti-PLA2R antibodies targeting different domains of PLA2R protein was associated with the disease severity and poor prognosis^31^. It will be interesting to investigate the association of *PLA2R1* and *HLA* genotypes with the types and titers of antibodies against different epitopes as well as with disease severity, treatment response and progression to renal failure.

Based on the findings in this study, further functional studies should also be designed to evaluate the roles of expression and missense changes in PLA2R in the context of pollutant exposures, binding to risk *HLA* alleles and recognition by autoantibodies. Studying multiple aspects of patients including transcriptomics, epigenomics, immune and renal profiles, and following them over the course of time, as has been done by Nephrotic Syndrome Study Network (NEPTUNE) cohort, would be helpful to fully understand the effects of genetic variants on downstream pathophysiological processes following immune provocation and the disease outcomes^32,33^. And finally, in order to get the full picture of risk signals in *PLA2R1* and *HLA* loci as well as to detect additional genetic susceptibility factors for IMN, we recommend large-scale multi-ethnic GWAS with accurate imputation, comparing local LD patterns, allele frequencies, disease models, and interaction with strong risk *HLA* alleles and comparing them among different populations.

## Methods

### Human subjects and samples

This study included 234 of idiopathic membranous nephropathy cases and 707 healthy controls. The initial genome-wide genotyping included 104 cases, of which 56 were collected by the Department of Hemodialysis and Apheresis of the University of Tokyo Hospital and 48 cases by the Department of Hematology, Nephrology and Rheumatology of Akita University and 419 healthy Japanese control samples which were collected at the Department of Human Genetics of the University of Tokyo. The diagnosis of IMN in cases was ensured by clinical features of generalized edema in the presence of heavy proteinuria (>3.5 g/day) and also by renal biopsy showing uniform, diffuse thickening of glomerular capillary wall without an increase in cellularity in light microscopic analysis of periodic acid-Schiff (PAS), periodic acid-methenamine-silver (PAM), and hematoxylin-eosin (HE) staining and transmission electron microscopy analysis. Granular staining pattern of complement C3 (C3) and IgG were confirmed at glomerular capillary wall in fluorescent microscopic analysis. Diseases that may cause secondary membranous nephropathy such as SLE, hepatitis, diabetes mellitus and cancers are screened by measuring Complete Blood Counts, ANA, HBV, HCV, HbA1c, Complements, Immunoglobulins, AST, ALT, Total Protein, Albumin, Total Cholesterol, Triglycerides and Electrolytes and checking medical records. Patients with such concomitant diseases were considered to be secondary membranous nephropathy cases and were excluded from the study. The control samples were from healthy volunteers.

The replication sample set contained 130 IMN cases collected by the BioBank Japan using the same criteria and 288 healthy control samples collected by the Department of Hemodialysis and Apheresis of the University of Tokyo Hospital. Control samples do not include individuals being diagnosed with any kidney disease, and all participants in this study are Japanese. Written informed consent was obtained from each participant before sample collection. The study was approved by the Ethical Committees at the Faculty of Medicine at the University of Tokyo, Akita University and the BioBank Japan, and all experiments in this study were performed in accordance with relevant guidelines and regulations.

### Genome-wide SNP genotyping, imputation and statistical analyses

Genome-wide SNP genotyping used Affymetrix Japonica genotyping array (Toshiba, Japan), which contains 659,636 SNPs based on the LD structure of 1,070 Japanese individuals with whole genome sequencing data^34^. Genotype calling was conducted in Axiom Analysis Suite v3.0.1. Genotype call rate for samples was set at 97% and 4 patients and 1 control were removed.

We removed 32,473 SNPs that were not assigned to be “Recommended” by the SNPolisher program in Axiom Analysis Suite, 43,967 SNPs with genotype call rate <99%, 115,513 SNPs with minor allele frequency (MAF) of <5% and 13,845 SNPs with Hardy-Weinberg equilibrium (HWE) *p*-value < 10^−3^ in both cases and controls. Identity-by-descent (IBD) test was run, and it detected 2 pairs of cases with >99% similar identity. One sample from each pair was removed. The quality control steps were performed using PLINK v1.9^35^.

Principal component analysis (PCA) was run for study samples together with samples from Hapmap 3 global populations^36^ using Genome-wide Complex Trait Analysis (GCTA) v1.02^37^. Five control samples located outside of the main Japanese cluster were excluded from further analysis (Supplementary Fig. S2). After these QC measures, 98 cases and 413 controls were left for further analysis.

SNP imputation was carried out using the whole genome sequence data of 2,048 Japanese individuals from the Tohoku Medical Megabank Organization (ToMMo) as reference panel. Phasing of the SNP genotypes into haplotypes was done using Eagle v2.3.5^38^, and imputation was performed using IMPUTE4 v1.0^39^. An info score of 0.5 was used for the quality control of imputed SNPs.

The genomic inflation factor (λ) was 1.03 after removing *HLA* and *PLA2R1* SNPs (Supplementary Fig. S3), suggesting no obvious population stratification. The Manhattan plot is shown in Supplementary Fig. S4.

Four *PLA2R1* SNPs were replicated in an independent sample set of 130 IMN patients and 288 healthy controls. The genotype data of SNPs reported in the previous Japanese study was provided by the authors of that study^7^, and SNPs not included in the previous study were genotyped by TaqMan assay.

Association analysis, conditional tests and haplotype analysis of *PLA2R1* SNPs were performed by using PLINK v1.07 (http://pngu.mgh.harvard.edu/purcell/plink/)^40^. The LD values between *PLA2R1* SNPs were calculated using Haploview v4.1^41^. The interaction between *PLA2R1* SNP haplotype and DRB1*15:01 was calculated in Microsoft Excel. The subgroup of patients and controls who were not recessive for the *PLA2R1* SNP haplotype and also negative for DRB1*15:01 is considered as the least risk group and compared with haplotype positive and DRB1*15:01 negative group, haplotype negative and DRB1*15:01 positive group and both haplotype and DRB1*15:01 positive group.

### Functional annotations

The expression quantitative trait loci (eQTL) information of non-coding SNPs was taken from the GTEx database^10^ (www.gtexportal.org/home/). The effects of missense SNPs on the PLA2R structure and function were checked using PolyPhen2 (http://genetics.bwh.harvard.edu/pph2/)^12^. The effect of amino acid changes caused by these missense SNPs on MHC-peptide binding with DRB1*15:01 protein was predicted by using the Immune Epitope Database (IEDB) analysis resource (http://tools.iedb.org/main/).

### HLA typing, imputation and statistical analyses

Classical HLA genotype data of *HLA-DRB1* and *HLA*-*DQB1* genes for 56 IMN cases from the University of Tokyo Hospital, 130 IMN cases from the replication stage and all control samples were provided by the authors of the previous study. For 48 IMN cases from Akita University, HLA imputation from the GWAS SNP data was performed by SNP2HLA v1.0.3^42^ using 419 healthy samples with Japonica array genotype data and HLA typing data for six *HLA* genes (*A, B, C, DPB1, DQB1* and *DRB1*) as reference.

To detect additional associated HLA alleles, relative predispositional effect (RPE) tests were applied to *HLA-DRB1* and *HLA-DQB1* loci. When a disease is associated with more than one allele of a gene, the strong association of one allele can create misleading deviations in the frequencies of the remaining alleles. RPE tests exclude the detected associated alleles and perform association tests of remaining alleles and the procedure is repeated to find the next largest RPE. This sequential process of identifying associated alleles and removing them is repeated until no significant overall deviation is observed. After excluding HLA-DRB1*15:01, RPE tests detected DRB1*14:54 and DRB1*11:01 sequentially in the *HLA-DRB1* locus. Similarly, in the *HLA-DQB1* locus, after excluding HLA- DQB1*06:02, DQB1*05:02 and DQB1*03:01 were significant. All three detected alleles of each locus were collapsed and projected into *DRB1* and *DQB1* collapsed risk alleles by treating each allele equally.

## Electronic supplementary material

Supplementary Data

## Data Availability

The top association results of *PLA2R1* and *HLA* regions and all the result data analyzed and discussed in this study are included in this published article and its Supplementary Information file.
