# Supplementary material for: Identification of a two-SNP PLA2R1 Haplotype and HLA-DRB1 Alleles as Primary Risk Associations in Idiopathic Membranous Nephropathy
Source: Sci Rep. 2018 Oct 22;8:15576. doi: 10.1038/s41598-018-33612-7 (PMC6197221; doi:10.1038/s41598-018-33612-7)
Supplement: Supplementary file 1 — Supplementary Data [file 41598_2018_33612_MOESM1_ESM.pdf]

Identification of a two-SNP *PLA2R1* Haplotype and *HLA-DRB1* Alleles as Primary Risk Associations in Idiopathic Membranous Nephropathy

Khun Zaw Latt, Kenjiro Honda, Myo Thiri, Yuki Hitomi, Yosuke Omae, Hiromi Sawai, Yosuke Kawai, Shunsuke Teraguchi, Kazuko Ueno, Masao Nagasaki, Akihiko Mabuchi, Hajime Kaga, Atsushi Komatsuda, Katsushi Tokunaga & Eisei Noiri

SUPPLEMENTARY FIGURES

Supplementary Figure S1. Expression quantitative trait loci (eQTL) associations of (a) rs4664308 and (b) rs3749119 in transformed fibroblasts from genotype-tissue expression (GTEx) database. The eQTL associations in transformed fibroblasts are the strongest among all tissue eQTL associations.

(a) (b)

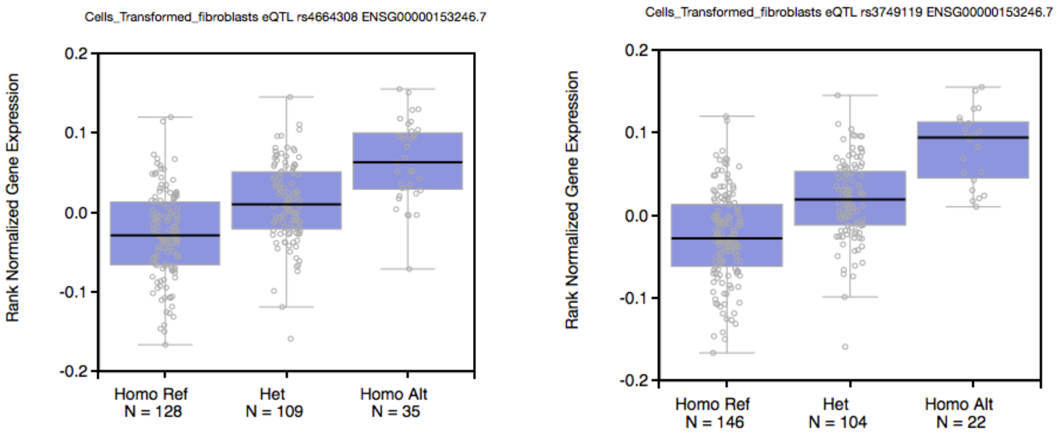

AA AG GG CC CT TT

The risk (major) alleles show lower level of gene expression.

Supplementary Figure S2. Principal Component Analysis (PCA). (a) with 3 global populations from Hapmap 3. (b) with only Chinese & Japanese samples from Hapmap 3.

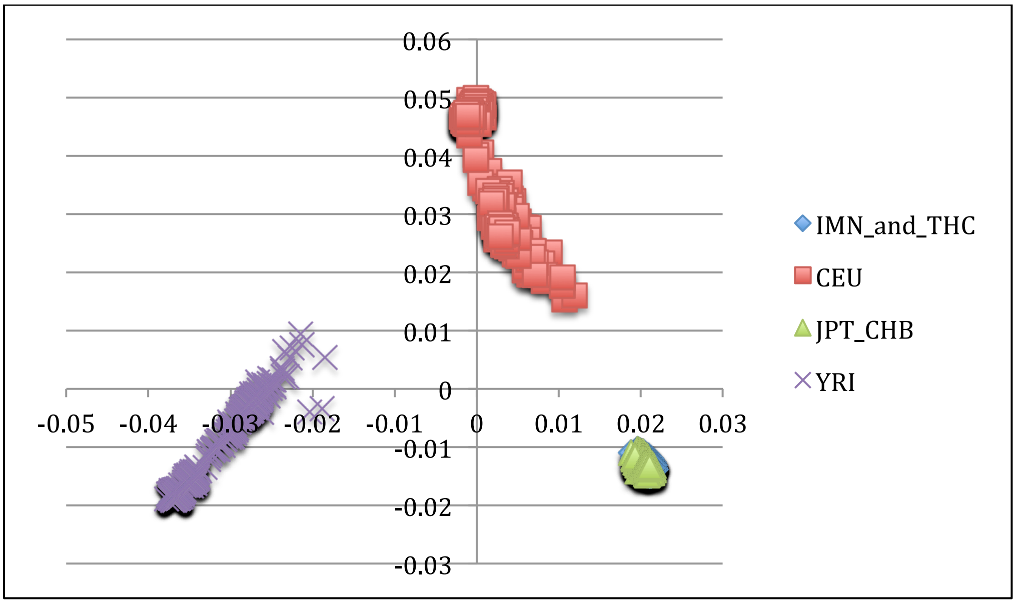

CEU = Northern European from Utah; YRI = Yoruba in Ibadan, Nigeria; CHB = Han Chinese in Beijing; JPT = Japanese from Tokyo.

(b)

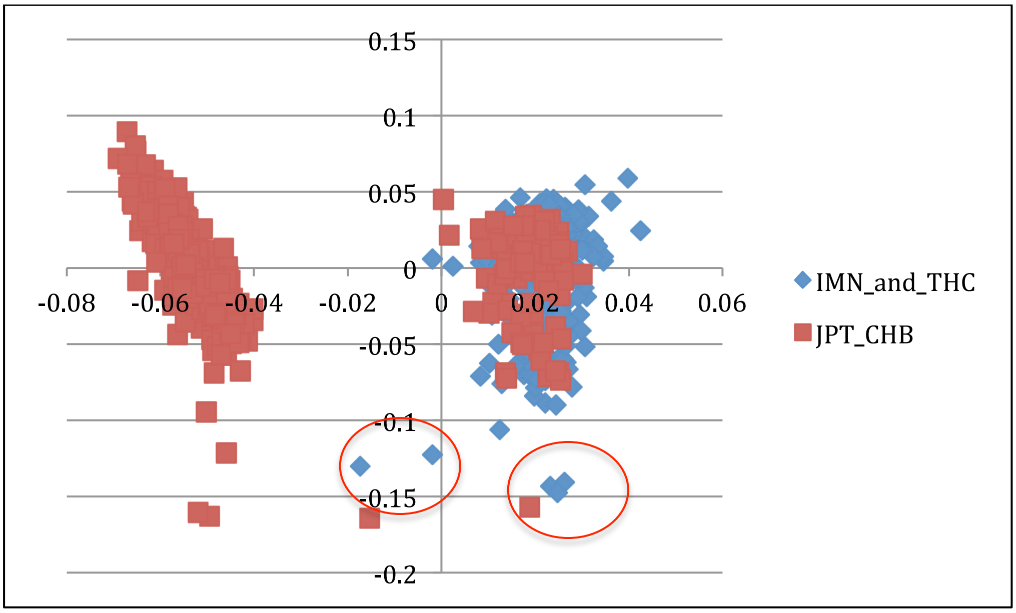

Five control samples that lie outside of main Japanese cluster were removed

Supplementary Figure S3. Quantile-quantile (Q-Q) plot of association results. X-axis = Expected –log P-values; Y-axis = Observed –log P values. (a) Before removing *HLA* and *PLA2R1* loci (b) After removing significant loci.
Genomic inflation factor (λ) = Observed/Expected median test statistics.

(a) (b)

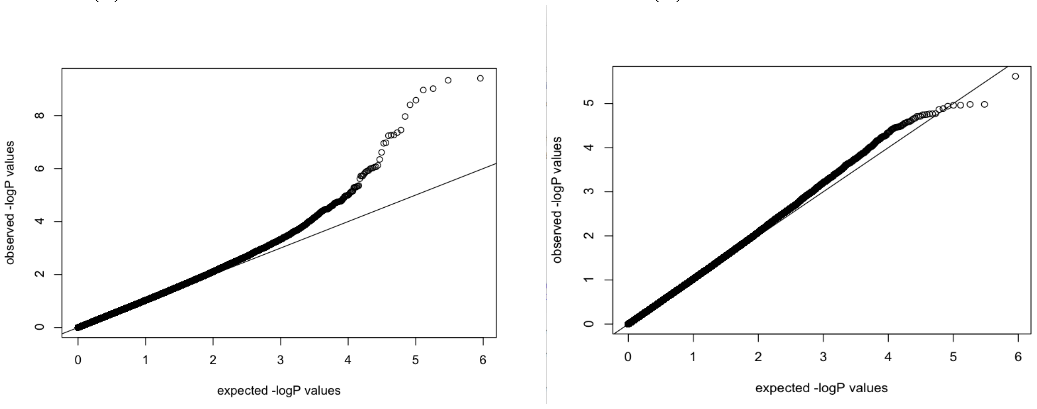

$\lambda = 1.032$ $\lambda = 1.030$

Supplementary Figure S4. Manhattan plot of whole genome SNP association results. X-axis = Chromosomes from 1 to 22. Y-axis = $-\log_{10}$ P-values of association results. Red horizontal line = genome-wide significance level (5×10^{-8}).

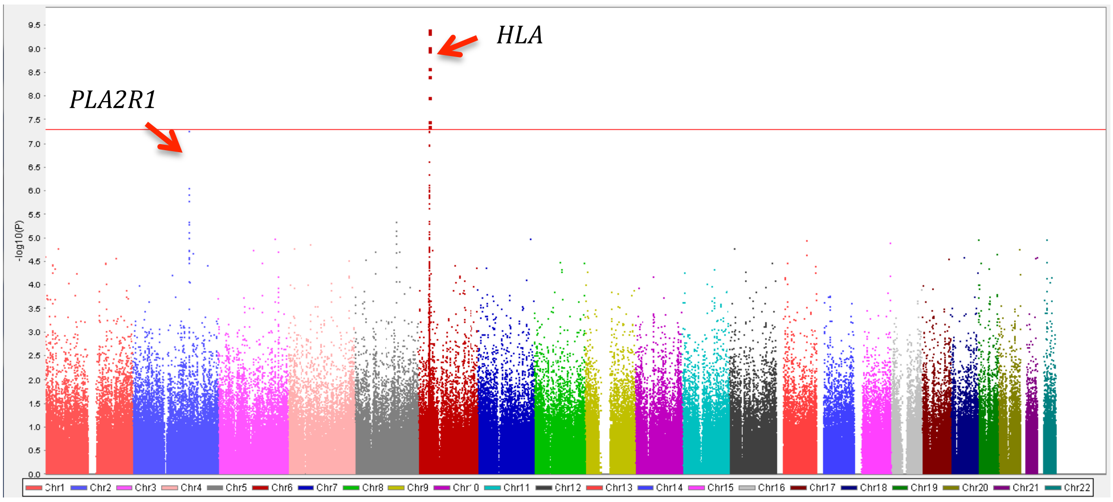

SUPPLEMENTARY TABLES

Supplementary Table S1. Results of imputation in *PLA2R1* region. (98 IMN vs. 413 controls).

SNP	Alleles (Minor/Major)	MAF (Case/Control)	OR (95% CI)	P	LD (r^2)
rs16844715	C/T	0.67/0.46	2.45 (1.75 - 3.4)	7.85×10^{-8}	
rs4665147	G/A	0.24/0.43	0.41 (0.28 - 0.59)	6.86×10^{-7}	1 with rs4664308
rs72621702	G/T	0.23/0.43	0.41 (0.28 - 0.59)	8.89×10^{-7}	
rs17831329	C/T	0.24/0.44	0.42 (0.29 - 0.6)	1.05×10^{-6}	
rs4664308	G/A	0.24/0.43	0.42 (0.29 - 0.60)	1.37×10^{-6}	
rs4665148	G/A	0.24/0.43	0.42 (0.29 - 0.60)	1.47×10^{-6}	
rs7563607	G/C	0.23/0.42	0.42 (0.29 - 0.6)	1.50×10^{-6}	
rs10929957	T/C	0.26/0.45	0.43 (0.30 - 0.61)	1.89×10^{-6}	

The signal remains essentially the same with genotyped results with rs16844715 being the top SNP and the second SNP, rs4665147 is in complete LD (r^2 = 1) with rs4664308, an intronic SNP and the European GWAS top hit.

Supplementary Table S2. Reciprocal conditional tests between rs4664308 and rs35771982. (222 IMN vs. 701 controls).

**Conditioning on rs4664308**					
rs35771982	A1	TEST	Samples	OR	P
	C	Additive	885	0.6205	0.08496
**Conditioning on rs35771982**					
rs4664308	A1	TEST	Samples	OR	P
	G	Additive	885	0.624	0.08553

In both cases, the remaining SNP showed no residual significance meaning that there is no significant independent effect between these SNPs.

Supplementary Table S3. Comparison of different models for SNP association (Upper) and comparison of SNP associations with haplotype association in recessive model (strongest) (Lower). (222 IMN vs. 701 controls).

SNP	Alleles (Major/Minor)	TEST (Risk Allele)	MAF (Case)	MAF (Control)	OR	P
rs35771982	G/C	Allelic	0.23	0.42	2.44	7.09×10^{-13}
		Recessive	0.39	0.67	3.23	1.41×10^{-13}
		Dominant	0.07	0.17	2.78	0.000251
rs4664308	A/G	Allelic	0.22	0.42	2.56	8.07×10^{-14}
		Recessive	0.38	0.66	3.13	2.12×10^{-13}
		Dominant	0.06	0.18	3.23	2.99×10^{-5}

Risk Haplotype	Test	Frequency (Case/Control)	OR (95% CI)	P
G-A	Haplotypic	0.765/0.55	2.68 (2.1 - 3.42)	3.76×10^{-15}
G-A	Recessive	0.61/0.296	3.66 (2.66 - 5.04)	9.65×10^{-16}

Supplementary Table S4. Interaction between SNP/Haplotype recessive form and *HLA-DRB1*15:01* positivity status. (+) means present; (-) means absent. (213 IMN vs. 610 controls).

SNP/Haplotype	SNP/Haplotype recessive status	DRB1*15:01 positivity status	Case (n=213)	%	Control (n=610)	%	OR (95% CI)	P
**rs4664308**	+	+	56	26.3%	25	4.1%	14.10 (8.12 – 24.49)	4.74×10^{-27}
	+	-	76	35.7%	175	28.7%	2.73 (1.84 – 4.05)	3.13×10^{-7}
	-	+	27	12.7%	68	11.2%	2.5 (1.47 – 4.25)	5.34×10^{-4}
	-	-	54	25.4%	340	55.7%	-	-
**rs35771982**	+	+	53	24.9%	26	4.3%	13.14 (7.58 – 22.77)	1.03×10^{-25}
	+	-	77	36.2%	169	27.7%	2.94 (1.98 – 4.35)	3.88×10^{-8}
	-	+	28	13.2%	67	11.0%	2.69 (1.59 – 4.56)	1.52×10^{-4}
	-	-	54	25.4%	348	57.1%	-	-
**rs4664308-rs35771982**	+	+	53	24.9%	21	3.4%	16.13 (9.05 – 28.78)	2.15×10^{-28}
	+	-	74	34.7%	155	25.4%	3.05 (2.06 – 4.53)	1.35×10^{-8}
	-	+	28	13.2%	71	11.6%	2.52 (1.5 – 4.24)	3.65×10^{-4}
	-	-	56	26.3%	358	58.7%	-	-

The association tests were performed by considering the sub-group negative for both SNP/Haplotype and *HLA-DRB1*15:01* (double-negative group) as baseline and comparing with all the remaining sub-groups. In haplotype/SNP positive sub-groups, haplotype risk shows stronger interaction with *HLA-DRB1*15:01* than individual SNP risks.

Supplementary Table S5. SNPs with strongest eQTL associations in 9 tissue types that are also in high LD with non-coding SNPs in current study.

**Tissue/Cell**	**SNP with strongest association**	**P**	**Effect Size**	**r^2 with rs4664308**
Adipose - Subcutaneous	rs16844715	1.60×10^{-11}	0.28	0.95
Cells - Transformed fibroblasts	rs58354887	3.10×10^{-26}	0.27	0.95
Colon - Sigmoid	rs877200	2.70×10^{-10}	0.41	1
Esophagus - Mucosa	rs58354887	1.00×10^{-27}	0.41	0.95
Muscle - Skeletal	rs58354887	7.30×10^{-18}	0.3	0.95
Spleen	rs877200	0.00001	0.24	1
Nerve - Tibial		3.70×10^{-16}	0.22	
Esophagus - Muscularis	rs3749119	1.90×10^{-11}	0.28	
Heart - Atrial Appendage		2.00×10^{-8}	0.29	

rs4664308 is in very high LD ($r^2 \geq 0.95$) with the strongest eQTL with positive effect sizes in six tissue/cell types. rs3749119 is the strongest eQTL SNP in three other tissues.

Supplementary Table S6. Prediction of PLA2R peptide binding with HLA-DRB1*15:01 protein by IEDB database.

Allele	Start	End	Peptide	Percentile rank	
				Risk	M292V-H300D
HLA-DRB1*15:01	278	292	FIREHMSSKTVEVWV	14.9	14.9
	279	293	IREHMSSKTVEVWVG	23.57	23.56
	280	294	REHMSSKTVEVWVGL	37.92	38.24
	281	295	EHMSSKTVEVWVGLN	53.07	56.32
	282	296	HMSSKTVEVWVGLNQ	48.03	50.77
	283	297	MSSKTVEVWVGLNQL	41.23	44.85
	284	298	SSKTVEVWVGLNQLD	39.94	44.75
	285	299	SKTVEVWVGLNQLDE	39.7	48.8
	286	300	KTVEVWVGLNQLDED	38.5	50.62
	287	301	TVEVWVGLNQLDEDA	41.41	56.13
	288	302	VEVWVGLNQLDEDAG	45.88	60.24
	289	303	EVWVGLNQLDEDAGW	54.62	68.18
	290	304	VWVGLNQLDEDAGWQ	63.27	77.33
	291	305	WVGLNQLDEDAGWQW	41.21	57.78
	292	306	VGLNQLDEDAGWQWS	42.27	59.01

Peptide column shows overlapping peptides of 15 amino acid residues from PLA2R protein sequence predicted for binding affinity with protein coded by DRB1*15:01. The lower the percentile rank, the higher is the binding affinity. The peptides containing 2 missense amino acid changes at positions 292 (rs3749117) and 300 (rs35771982) are predicted to have lower percentile ranks.

Supplementary Table S7. Top associated SNPs from HLA region with $P < 10^{-5}$ in the initial genome-wide analysis. (98 IMN vs. 413 controls).

CHR	SNP	Alleles (Minor/Major)	MAF (Case/Control)	OR (95% CI)	1/OR	P
6	rs2524075	T/C	0.14/0.31	0.37 (0.24 - 0.56)	2.74	2.41×10^{-6}
	rs429608	A/G	0.25/0.12	2.38 (1.62 - 3.51)	0.42	7.30×10^{-6}
	rs415929	C/T	0.18/0.36	0.39 (0.27 - 0.58)	2.54	1.90×10^{-6}
	rs75117278	G/A	0.20/0.09	2.72 (1.78 - 4.16)	0.37	1.89×10^{-6}
	rs386554868	C/T	0.58/0.39	2.20 (1.60 - 3.02)	0.45	7.59×10^{-7}
	rs3135365	C/A	0.31/0.16	2.35 (1.65 - 3.36)	0.42	1.43×10^{-6}
	rs9469110	T/G	0.31/0.13	3.11 (2.15 - 4.49)	0.32	3.93×10^{-10}
	rs9501626	A/C	0.30/0.13	3.03 (2.10 - 4.39)	0.33	1.09×10^{-9}
	rs3129878	C/A	0.31/0.51	0.44 (0.32 - 0.62)	2.27	9.49×10^{-7}
	rs9268671	A/G	0.42/0.24	2.33 (1.68 - 3.23)	0.43	2.45×10^{-7}
	rs9268834	A/C	0.23/0.4	0.45 (0.31 - 0.64)	2.23	9.69×10^{-6}
	rs6923504	G/C	0.46/0.25	2.68 (1.94 - 3.70)	0.37	9.62×10^{-10}
	rs6903608	C/T	0.48/0.26	2.60 (1.89 - 3.58)	0.38	2.64×10^{-9}
	rs9268853	C/T	0.22/0.39	0.43 (0.30 - 0.63)	2.31	5.01×10^{-6}
	rs9268877	A/G	0.52/0.32	2.33 (1.70 - 3.20)	0.43	1.06×10^{-7}
	rs58667488	C/A	0.15/0.31	0.38 (0.25 - 0.58)	2.61	4.38×10^{-6}
	rs9268923	T/C	0.22/0.39	0.43 (0.30 - 0.63)	2.31	5.01×10^{-6}
	rs2395185	T/G	0.22/0.39	0.44 (0.30 - 0.63)	2.29	7.32×10^{-6}
	rs9268978	A/G	0.24/0.08	3.52 (2.33 - 5.33)	0.28	4.67×10^{-10}
	rs9269081	A/C	0.44/0.24	2.45 (1.77 - 3.38)	0.41	3.52×10^{-8}
	rs9269114	A/G	0.45/0.25	2.55 (1.84 - 3.53)	0.39	1.08×10^{-8}
	rs5020946	T/G	0.27/0.46	0.43 (0.30 - 0.61)	2.33	9.78×10^{-7}
	rs12191360	C/A	0.54/0.33	2.40 (1.74 - 3.30)	0.42	5.40×10^{-8}
	rs12660447	A/G	0.57/0.34	2.55 (1.85 - 3.50)	0.39	3.92×10^{-9}
	rs13194665	G/A	0.28/0.47	0.44 (0.31 - 0.61)	2.30	1.29×10^{-6}
	rs9271191	T/C	0.34/0.17	2.58 (1.82 - 3.65)	0.39	4.38×10^{-8}
	rs3135005	A/G	0.34/0.17	2.52 (1.78 - 3.56)	0.40	1.12×10^{-7}
	rs9271366	G/A	0.32/0.17	2.36 (1.66 - 3.36)	0.42	1.02×10^{-6}
	rs9271493	A/G	0.54/0.35	2.23 (1.63 - 3.05)	0.45	4.53×10^{-7}
	rs9469220	G/A	0.46/0.3	2.04 (1.49 - 2.81)	0.49	8.33×10^{-6}
	rs2647012	T/C	0.35/0.19	2.33 (1.65 - 3.28)	0.43	8.63×10^{-7}
	rs2647046	A/C	0.35/0.19	2.26 (1.61 - 3.19)	0.44	1.89×10^{-6}
	rs3135002	A/C	0.23/0.09	2.98 (1.98 - 4.49)	0.34	5.75×10^{-8}
	rs2856717	A/G	0.35/0.19	2.30 (1.63 - 3.24)	0.43	1.23×10^{-6}

CHR = Chromosome; SNP = single nucleotide polymorphism, MAF = minor allele frequency.

Supplementary Table S8. Results of SNP imputation in *HLA* region. (98 IMN vs. 413 controls).

SNP	Alleles (Minor/Major)	MAF (Case/Control)	OR (95% CI)	P
rs5026743	G/T	0.5/0.25	2.97 (2.15 - 4.11)	1.56×10^{-11}
rs9271147	T/C	0.25/0.08	3.71 (2.45 - 5.60)	6.63×10^{-11}
rs9269110	A/C	0.47/0.25	2.72 (1.96 - 3.77)	9.27×10^{-10}
rs77709710	A/T	0.22/0.074	3.6 (2.34 - 5.54)	9.82×10^{-10}
rs4959100	T/C	0.31/0.13	3.05 (2.11 - 4.42)	1.14×10^{-9}
rs9469110	T/G	0.31/0.13	3.05 (2.11 - 4.42)	1.14×10^{-9}
rs9501626	A/C	0.31/0.13	3.05 (2.11 - 4.42)	1.14×10^{-9}
rs2027856	A/G	0.31/0.13	3.05 (2.11 - 4.42)	1.14×10^{-9}
rs9268880	T/G	0.46/0.24	2.67 (1.93 - 3.71)	1.60×10^{-9}
rs6923504	G/C	0.46/0.25	2.67 (1.92 - 3.69)	1.79×10^{-9}
rs6919855	C/T	0.46/0.25	2.67 (1.92 - 3.69)	1.79×10^{-9}
rs9268882	T/C	0.46/0.25	2.67 (1.92 - 3.69)	1.79×10^{-9}

rs9271147 located between *HLA-DRB1* and *HLA-DQB1* showed the strongest association.

Supplementary Table S9. Association result of rs2187668 in genotyped samples. (180 IMN vs. and 701 controls).

CHR	SNP	Alleles (Minor/Major)	MAF (Case/Control)	OR (95% CI)	P
6	rs2187668	T/C	0.04/0.0333	1.17 (0.64 – 2.16)	0.61

rs2187668 is a well-known tag SNP for *HLA-DRB1*03:01* and the absence of that allele in the Japanese population explains the lack of association of this SNP.

Supplementary Table S10. The association results of *HLA-DRB1* and *HLA-DQB1* collapsed alleles in allelic, dominant and recessive models. (222 IMN vs. 701 controls)

**Model**	**Allele**	**Alleles (Minor/Major)**	**Case MAF**	**Control MAF**	**OR (95% CI)**	**P**
Allelic	DRB1_collapsed_risk	P/A	0.31	0.14	2.82 (2.17 – 3.66)	1.57×10^{-15}
	DQB1_collapsed_risk	P/A	0.39	0.21	2.41 (1.9 – 3.05)	2.02×10^{-13}
Dominant	DRB1_collapsed_risk	P/A	0.56	0.26	3.64 (2.63 – 5.04)	1.13×10^{-15}
	DQB1_collapsed_risk	P/A	0.65	0.38	3.06 (2.21 – 4.24)	5.75×10^{-12}
Recessive	DRB1_collapsed_risk	P/A	0.065	0.02	3.51 (1.6 – 7.71)	0.00094
	DQB1_collapsed_risk	P/A	0.13	0.042	3.42 (1.95 – 5.97)	5.93×10^{-6}

The collapsed risk alleles are most strongly associated in the dominant model.

Supplementary Table S11. Interaction between the positivity status of *HLA-DRB1*14:54*, *HLA-DRB1*11:01* and DRB1 collapsed allele and *PLA2R1* haplotype recessive form. (+) means present; (-) means absent. (211 IMN vs. 605 controls).

DRB1 alleles	*PLA2R1* Haplotype recessive status	DRB1 alleles positivity status	Case (n=211)	%	Control (n=605)	%	OR (95% CI)	P
*DRB1*14:54*	+	+	14	6.6%	14	2.3%	5.19 (2.38 - 11.32)	5.83×10^{-6}
	+	-	113	53.6%	162	26.8%	3.62 (2.57 - 5.09)	2.85×10^{-14}
	-	+	6	2.8%	24	4.0%	1.30 (0.51 - 3.28)	0.77
	-	-	78	37.0%	405	66.9%	-	-
*DRB1*11:01*	+	+	11	5.2%	8	1.3%	7.44 (2.9 - 19.12)	7.38×10^{-6}
	+	-	116	55.0%	168	27.8%	3.74 (2.66 - 5.26)	6.37×10^{-15}
	-	+	9	4.3%	23	3.8%	2.12 (0.94 - 4.76)	0.11
	-	-	75	35.5%	406	67.1%	-	-
DRB1_collapsed_risk	+	+	74	35.1%	43	7.1%	12.99 (7.92 - 21.3)	1.66×10^{-29}
	+	-	53	25.1%	133	22.0%	3.01 (1.91 - 4.73)	9.57×10^{-7}
	-	+	42	19.9%	112	18.5%	2.83 (1.75 - 4.57)	1.25×10^{-5}
	-	-	42	19.9%	317	52.4%	-	-

Both *DRB1*14:54* and *DRB1*11:01* showed interaction effects with *PLA2R1* haplotype, albeit more weakly than *DRB1*15:01*. The interaction effect of DRB1 collapsed risk becomes weaker than that of *DRB1*15:01* because *DRB1*15:01* effect was diluted by the weaker effects of these two alleles to become DRB1 collapsed risk allele.

Supplementary Table S12. SNPs in high LD (r^2 > 0.9) with rs4664308 in Asian LD from HaploReg v4.1, their LD values with this SNP, allele frequencies in different populations, functional annotations and functional genomic data.

Chr	variant	LD (r^2)	GENCODE genes	dbSNP func annot	ASN freq	EUR freq	Enhancer histone marks
2	rs35362017	0.92	PLA2R1	intronic	0.33	0.41	Huvec
2	rs56297666	0.91	PLA2R1	intronic	0.32	0.42	
2	rs17341301	0.92	PLA2R1	intronic	0.33	0.42	
2	rs34248629	0.92	PLA2R1	intronic	0.33	0.42	
2	rs877200	0.92	PLA2R1	intronic	0.33	0.42	
2	rs2175416	0.92	PLA2R1	intronic	0.33	0.42	
2	rs1511218	0.92	PLA2R1	intronic	0.33	0.42	
2	rs10929965	0.92	PLA2R1	intronic	0.33	0.42	Huvec
2	rs6719686	0.92	PLA2R1	intronic	0.33	0.42	Huvec, NHEK
2	rs10929966	0.92	PLA2R1	intronic	0.33	0.42	Huvec, NHEK
2	rs17831161	0.92	PLA2R1	intronic	0.33	0.42	NHEK, Huvec
2	rs62175515	0.92	PLA2R1	intronic	0.33	0.42	NHEK, Huvec
2	rs7563607	0.94	PLA2R1	intronic	0.33	0.42	Huvec
2	rs56293836	0.95	PLA2R1	intronic	0.33	0.42	
2	rs17831191	0.95	PLA2R1	intronic	0.33	0.42	
2	rs62175517	0.95	PLA2R1	intronic	0.33	0.42	H1, Huvec
2	rs925410	0.95	PLA2R1	intronic	0.33	0.42	H1, NHEK
2	rs6759836	0.95	PLA2R1	intronic	0.33	0.42	H1
2	rs17831251	0.95	PLA2R1	intronic	0.33	0.42	
2	rs6707458	0.92	PLA2R1	intronic	0.34	0.42	
2	rs62175518	0.92	PLA2R1	intronic	0.33	0.43	
2	rs17241792	0.98	PLA2R1	intronic	0.34	0.42	
2	rs17831329	1	PLA2R1	intronic	0.34	0.42	
2	rs4665147	1	PLA2R1	intronic	0.34	0.42	NHEK
2	rs72621702	0.95	PLA2R1	intronic	0.33	0.03	HMEC, Huvec, NHEK
2	rs4664308	1	PLA2R1	intronic	0.34	0.42	4 cell types
2	rs4665148	1	PLA2R1	intronic	0.34	0.42	HMEC, Huvec
2	rs17241973	0.95	PLA2R1	intronic	0.34	0.42	
2	rs3749119	0.94	PLA2R1	5'-UTR	0.33	0.34	
2	rs141127009	0.91	2.8kb 5' of PLA2R1		0.32	0.41	
2	rs77917627	0.88	3.6kb 5' of PLA2R1		0.31	0.03	
2	rs58354887	0.91	8kb 5' of PLA2R1		0.32	0.41	
2	rs6744567	0.9	15kb 5' of PLA2R1		0.32	0.41	Huvec
2	rs7593593	0.9	15kb 5' of PLA2R1		0.32	0.41	Huvec

The causal regulatory SNP is likely to be one of these SNPs. However, the very high LD among these SNPs makes it extremely difficult to pinpoint the causal SNP confidently by statistical methods alone.

Supplementary Table S13. SNPs in high LD ($r^2 > 0.9$) with rs35771982 in Asian LD from HaploReg v4.1, their LD values with this SNP, allele frequencies in different populations, functional annotations and functional genomics data.

Chr	variant	LD (r^2)	GENCODE genes	dbSNP func annot	ASN freq	EUR freq	Enhancer histone marks
2	rs55668231	0.97	PLA2R1	intronic	0.35	0.48	
2	rs35771982	1	PLA2R1	missense	0.35	0.48	NHLF, HSMM
2	rs3749117	1	PLA2R1	missense	0.35	0.49	NHLF, HSMM
2	rs66667042	0.99	PLA2R1	intronic	0.35	0.48	
2	rs3792192	0.99	PLA2R1	intronic	0.35	0.48	
2	rs17830755	0.99	PLA2R1	intronic	0.35	0.48	
2	rs4665138	0.99	PLA2R1	intronic	0.35	0.48	Huvec, HSMM, NHLF
2	rs4665139	0.97	PLA2R1	intronic	0.35	0.48	Huvec, HSMM, NHLF
2	rs4665140	0.99	PLA2R1	intronic	0.35	0.48	Huvec, HSMM, NHLF
2	rs4665141	0.99	PLA2R1	intronic	0.35	0.48	Huvec, NHLF, HSMM
2	rs17241282	0.99	PLA2R1	intronic	0.35	0.42	Huvec, NHLF, HSMM
2	rs58040648	0.99	PLA2R1	intronic	0.35	0.48	Huvec, HSMM
2	rs59137532	0.92	PLA2R1	intronic	0.34	0.02	Huvec, HSMM
2	rs17830904	0.98	PLA2R1	intronic	0.35	0.48	Huvec
2	rs17830940	0.95	PLA2R1	intronic	0.35	0.48	Huvec
2	rs62175487	0.98	PLA2R1	intronic	0.35	0.48	
2	rs62175488	0.98	PLA2R1	intronic	0.35	0.48	
2	rs6722275	0.98	PLA2R1	intronic	0.35	0.48	
2	rs200942289	0.95	PLA2R1	intronic	0.36	0.49	
2	rs66484345	0.98	PLA2R1	intronic	0.35	0.48	Huvec

rs35771982 and rs3749117 are the only two missense SNPs in this list of SNPs in high LD. They are also in complete LD making it difficult to distinguish which one is real primary risk or if both of them are necessary to confer disease risk.
